# *Chlorella vulgaris* as a Livestock Supplement and Animal Feed: A Comprehensive Review

**DOI:** 10.3390/ani15060879

**Published:** 2025-03-19

**Authors:** Ishaya Usman Gadzama, Saraswati Ray, René Méité, Isaac Maina Mugweru, Takudzwa Gondo, Md Atikur Rahman, Md Rahat Ahmad Redoy, Md Fazle Rohani, Ahmed Eid Kholif, Md Salahuddin, Andre F. Brito

**Affiliations:** 1 School of Agriculture and Food Sustainability, University of Queensland, Gatton, QLD 4343, Australia; 2 School of Environmental and Rural Science, Faculty of Science, Agriculture, Business and Law (SABL), University of New England, Armidale, NSW 2351, Australia; 3 Leibniz Centre for Agricultural Landscape Research (ZALF), Eberswalder Straße 84, 15374 Müncheberg, Germany; rene.meite@zalf.de; 4 Albrecht Daniel Thaer-Institute of Agricultural and Horticultural Sciences, Department of Agricultural Economics, Humboldt University of Berlin, 10117 Berlin, Germany; 5 Department of Animal Sciences, College of Agriculture and Natural Resources (COANRE), Jomo Kenyatta University of Agriculture and Technology, Nairobi 002001, Kenya; mugwerumainake@gmail.com; 6 Department of Animal and Poultry Science, University of Saskatchewan, Saskatoon, SK S7N 5A8, Canada; takudzwagondo7@gmail.com; 7 Department of Agriculture, Nutrition, and Food Systems, University of New Hampshire, Durham, NH 03824, USA; mdatikur.rahman@unh.edu (M.A.R.); andre.brito@unh.edu (A.F.B.); 8 Department of Animal Science, University of Connecticut, Storrs, CT 06269, USA; rahat@uconn.edu; 9 Nutrition and Seafood Laboratory (NuSea.Lab), School of Life and Environmental Sciences, Deakin University, Queenscliff, VIC 3225, Australia; rohani_aq@bau.edu.bd; 10 Department of Aquaculture, Bangladesh Agricultural University, Mymensingh 2202, Bangladesh; 11 Department of Animal Science, North Carolina A&T State University, Greensboro, NC 27411, USA; aekholif@ncat.edu; 12 Poultry Center, Cooperative Agricultural Research Center, Prairie View A&M University, Prairie View, TX 77446, USA; mdsalahuddin@pvamu.edu

**Keywords:** microalgae, *Chlorella vulgaris*, rumen fermentation, antioxidants, immune response, nutrient digestibility, growth performance, milk production, egg quality, meat quality, sustainable agriculture

## Abstract

This review explores the potential of *Chlorella vulgaris* (CLV) as an alternative supplement in animal feed. CLV is rich in essential nutrients including fatty acids, amino acids, vitamins, and minerals, as well as bioactive compounds such as antioxidants, which contribute to its health-promoting properties. The nutritional composition of CLV can vary depending on factors such as cultivation methods, nutrient availability, light intensity, temperature, water pH, strain, and processing techniques. The rigid cell wall of the microalga limits nutrient accessibility, particularly in monogastric animals. However, processing techniques such as enzymatic treatments can disrupt the cell wall, enhancing nutrient bioavailability and improving its utility as a feed ingredient. Research across livestock species has demonstrated the positive effects of CLV supplementation. For instance, CLV has improved milk production and composition in ruminants, modulated rumen microbiota, enhanced lamb growth, and elevated blood immunoglobulin levels. Moreover, the impact of CLV on ruminal fermentation is dose-dependent, with higher inclusion rates exhibiting more pronounced effects, and it may also play a role in mitigating methane emissions. In poultry, CLV supplementation leads to better growth, feed conversion ratios, immune responses, and meat and egg quality. Similarly, studies on pigs suggest that CLV can benefit immune response and fatty acid profiles, while in rabbits, CLV has been found to reduce oxidative stress and improve immune responses. Additionally, CLV has shown promise in aquaculture, improving feed utilization, immunity, and disease resistance in various fish species. While CLV shows considerable potential, the variability in animal responses and the need for optimized inclusion levels necessitate further species-specific research to elucidate the long-term implications of its inclusion in animal diets.

## Simple Summary

*Chlorella vulgaris* (CLV) is a green microalga with significant potential as a sustainable animal feed supplement due to its rich nutritional composition. The nutritional profile of CLV, which includes fatty acids, amino acids, vitamins, and minerals, can vary significantly depending on cultivation conditions such as nutrient availability, light intensity, temperature, water pH, and strain. Studies across various livestock species, such as ruminants, poultry, pigs, rabbits, and fish, suggest that CLV supplementation can lead to several benefits. These benefits include improved growth performance, enhanced nutrient digestibility, better product quality, and overall improvements in animal health and welfare. However, the effects of CLV supplementation are dose-dependent and can vary across different animal species. Therefore, determining optimal inclusion levels is crucial, and further species-specific research is necessary to fully understand the long-term implications of CLV in animal diets.

## 1. Introduction

**Figure 1:**
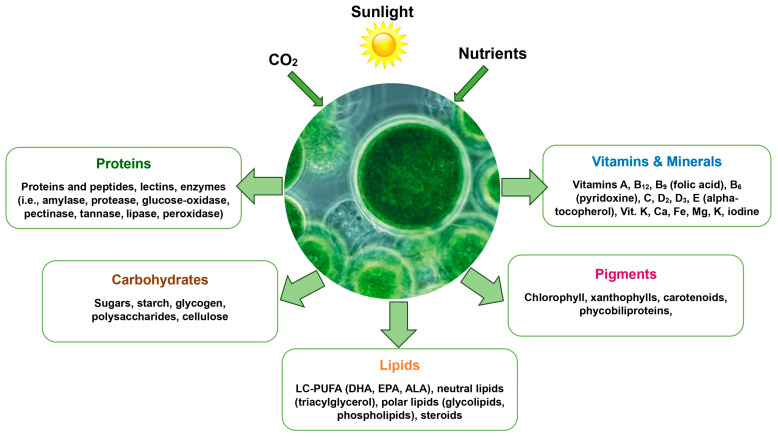
The nutritional value of *Chlorella vulgaris* as an animal feed supplement. Adapted from Saadaoui et al. [13].

Global demand for meat, milk, and eggs is projected to surge by 60–70% by 2050, driven by population growth, rising incomes, and dietary shifts in developing countries. Meeting this growing demand requires more efficient and environmentally sustainable methods of livestock feeding, as traditional feed production is often resource-intensive and competes with other agricultural needs [1,2]. To address these challenges, feed supplements have been widely incorporated into animal diets to optimize health, growth, reproduction, and overall production efficiency [3,4,5]. Among these supplements, those derived from the green microalgae *Chlorella vulgaris* (CLV) are gaining recognition for their multifaceted benefits, including high nutrient density and the potential to replace conventional feed ingredients [3,4,5,6]. For instance, microalgae can serve as alternatives to synthetic vitamins, amino acids, and antibiotics in conventional feeds, enhancing nutrient bioavailability and supporting animal health without contributing to antimicrobial resistance [4,5,6]. *Chlorella vulgaris* stands out as a promising alternative due to its high nutritional value and potential benefits for animal performance, product quality, and welfare [3,4,5,6]. Studies demonstrate that CLV supplementation enhances innate immunity in poultry, improves omega-3 fatty acid profiles in ruminant-derived products, and increases antioxidant levels in eggs and milk, positioning it as a multifunctional supplement for diverse livestock systems [4,5,6]. Microalgae, including CLV, are photosynthetic organisms that thrive in either photoautotrophic (utilizing sunlight, CO_2_, and water) or heterotrophic environments [5,6]. They require minimal resources to convert into nutrient-rich biomass [7,8,9]. This biomass is packed with essential nutrients such as fatty acids, amino acids, carotenoids, carbohydrates, pigments, vitamins, minerals, and antioxidants, making it a highly valuable feed ingredient (Figure 1) [10,11,12,13,14].

The cultivation of CLV has a relatively lower environmental impact compared to traditional agriculture. It requires less land and can be grown in controlled environments, reducing the need for pesticides and fertilizers [15]. CLV is known for its rapid growth, with a doubling time of less than 20 h [16], allowing for efficient and potentially large-scale production. This suggests that CLV can contribute to sustainable animal feed production by offering a nutrient-rich alternative to traditional feed sources while requiring fewer resources and minimizing competition with other agricultural sectors.

Studies suggest CLV supplementation can improve animal growth performance, nutrient digestibility, product quality, and overall health and welfare. This paper discusses the potential implications of dietary CLV supplementation as a sustainable animal feed. It examines the nutrient composition of CLV and reviews the results of studies conducted both in living animals and in laboratory settings using rumen fermentation techniques. The research explores how CLV impacts animal physiology and performance, and its implications for livestock production, including its potential to improve nutrient breakdown and mitigate methane emissions, which is significant for environmental sustainability.

## 2. Materials and Methods

### 2.1. Literature Search

A systematic and comprehensive literature search was conducted to evaluate the potential of *Chlorella vulgaris* (CLV) as a sustainable and effective animal feed supplement. The search spanned multiple academic databases, including Google Scholar, ScienceDirect, Scopus, PubMed, and Web of Science, to ensure a broad and inclusive coverage of relevant studies. The primary objective was to identify peer-reviewed, primary research articles that provided original experimental data on the efficacy of CLV in animal diets. This focus on primary studies was critical to ensure methodological rigor, data accuracy, and the ability to directly assess outcomes such as methane reduction, growth performance, nutrient digestibility, and physiological effects in animals. Both *in vitro* and *in vivo* studies were included to evaluate the reproducibility of findings and their relevance to real-world farming scenarios. Peer-reviewed journal articles formed the core of the synthesis. However, conference papers were occasionally referenced to capture emerging insights, provided they offered novel and credible data.

### 2.2. Searching Criteria

The search strategy employed a combination of keywords and phrases related to *Chlorella vulgaris* and its application in animal nutrition. Key terms included “*Chlorella vulgaris* nutrient composition”, “*Chlorella vulgaris* protein content”, “*Chlorella vulgaris* fatty acid profile”, “*Chlorella vulgaris* methane reduction”, “*Chlorella vulgaris* ruminal fermentation”, “*Chlorella vulgaris* animal growth performance”, and “*Chlorella vulgaris* immune response”, among others. Boolean operators (AND/OR) were used to refine the search and maximize the retrieval of relevant studies. Additional filters were applied to focus on studies involving ruminants, poultry, aquaculture, swine, and rabbits, aligning within the scope of this review. To ensure comprehensiveness, no restrictions were placed on publication years, allowing the inclusion of foundational studies as well as recent advancements. Reference lists of retrieved articles were manually screened to identify additional relevant publications, which were then sourced from the aforementioned databases. Non-academic sources, such as commercial websites or unverified reports, were excluded to maintain scientific rigor. Studies focusing on other strains of *Chlorella* or non-feed applications of algae were also omitted to ensure a focused and high-quality synthesis.

## 3. The Evolution and Global Trends of *Chlorella vulgaris* Production

*Chlorella vulgaris*, a single-celled green microalga, holds a significant place in evolutionary history as one of Earth’s most ancient life forms, with origins dating back to the Precambrian epoch approximately 2.5 billion years ago [10,17]. The name ‘Chlorella’ derives from the Greek ‘chlorós’, meaning ‘green’, and the diminutive Latin suffix ‘ella,’ meaning ‘small’ [10]. Discovered in 1890 by Dutch microbiologist and botanist Martinus Willem Beijerinck, CLV is characterized by a robust cell wall, ranging from 100 to 200 nm in thickness, providing substantial mechanical and chemical defense [10]. Taxonomically, CLV is classified as follows: Domain: *Eukaryota*; Kingdom: *Protista*; Phylum: *Chlorophyta*; Class: *Trebouxiophyceae*; Order: *Chlorellales*; Family: *Chlorellaceae*; Genus: *Chlorella*; and Species: *Chlorella vulgaris* [10].

The production of CLV has evolved significantly since its discovery. Large-scale cultivation began in the early 1960s in Japan, where it became a popular food source [18]. Today, over 70 companies worldwide are involved in its production (Table 1). Taiwan leads in production, with the Taiwan Chlorella Manufacturing Company in Taipei producing over 400 tons of dried biomass annually. Other major producers include Japan and Germany, with the Klötze facility in Germany producing 130 to 150 tons of dry biomass annually [8]. By 2009, annual production of Chlorella biomass had surpassed 2000 metric tons (dry weight) [14,19,20].

## 4. Chemical Composition of *Chlorella vulgaris*

The chemical composition of CLV shows significant variations based on the growth conditions used in different studies (Table 2). This variation is evident in components such as dry matter (DM), organic matter (OM), crude protein (CP), ether extract (EE), neutral detergent fiber (NDF), acid detergent fiber (ADF), and ash content. Additional components, such as starch, crude fiber (CF), available carbohydrate (CHO), and non-structural carbohydrate (NSC), were included to understand the nutritional value and potential applications of CLV as animal feed.

The reported DM values range from 92.7% to 96.1%, indicating a relatively consistent DM content under different growth conditions. CLV demonstrates a high CP content, ranging from 22.7% to 67.7% of dry matter, reinforcing its potential as a valuable protein source for food and feed applications [21,50]. Similarly, the EE, representing lipid content, shows considerable variation, ranging from 2.4% to 14.2% [21,27]. This variation indicates the impact of growth conditions on lipid accumulation in CLV, potentially affecting its energy content and fatty acid profiles.

**Table 2:** Chemical composition (% DM) of *Chlorella vulgaris* under different growth conditions.

DM	OM	CP	EE	NDF	ADF	Ash	Energy	Reference
93.1	81.3	42.8	-	32.2	19.4	11.8	-	[3]
93.1	-	46.0	9.4	-	-	12.7	4586 ^a^	[51]
95.5	-	22.7	14.2	-	-	-	434 ^b^	[21]
93.2	94.2	57.9	13.9	11.8	4.3	-	-	[52]
95.4	-	48.4	9.2	20.2	-	11.1	18.8 ^c^	[44]
94.6	-	60.8	9.5	0.0	-	5.7	-	[53]
94.2	90.5	51.5	12.2	9.2	-	-	-	[22,23]
92.7	84.8	67.7	10.5	12.8	4.2	-	-	[50]
94.6	-	60.6	12.8	-	-	4.5	-	[54]
NR	-	25.0	2.4	-	-	-	-	[27]
96.1	-	47.8	13.3	-	-	6.3	1427 ^d^	[55]
**Others**	**Amount**							**Reference**
Starch	4.4							[44]
	4.3							[53]
CF	8.8							[3]
	13.0							[54]
	5.4							[27]
CHO	8.1							[55]
NSC	10.6							[52]
	11.9							[22,23]

- = not reported; ^a^ GE, gross energy (Cal/g); ^b^ Kcal; ^c^ GE, gross energy (MJ/kg DM); ^d^ kJ; DM, dry matter; OM, organic matter; CP, crude protein; EE, ether extract; NDF, neutral detergent fiber; ADF, acid detergent fiber; CF, crude fiber; CHO, available carbohydrate; NSC, non-structural carbohydrate.

The fiber content, reported as NDF and ADF, also shows variability. NDF values range from 0.0% to 32.2%, while ADF values range from 4.2% to 19.4%, which may have implications for gut health and digestion in animals. Ash content, indicative of mineral content, ranges from 4.5% to 12.7%. The reported energy values fluctuate, likely due to variations in measurement methods. Data on carbohydrate composition are more limited but highlight the presence of various carbohydrates, including starch, CF, available carbohydrates (CHO), and non-structural carbohydrates (NSC). Starch content varies from 4.3% to 4.4%, and CF ranges from 5.4% to 13.0%. These components are crucial for understanding the potential digestibility and energy value of CLV as an animal feed supplement.

The observed variations in the nutritional content of CLV can be attributed to several factors, including differences in cultivation methods, nutrient availability, light intensity, temperature, pH, and processing techniques [10,56,57]. Although the specific growth conditions were not explicitly stated in this paper, we inferred that these conditions play a significant role in shaping the chemical composition of this microalga. Additionally, variations in sample preparation, extraction techniques, and analytical instruments can lead to differences in measured nutrient levels. From an animal nutrition perspective, CLV has promising potential as a feed component. However, research is necessary to determine the ideal conditions for maximizing specific nutrient levels and to optimize processing techniques to ensure consistent and desirable nutritional content for effective utilization as an animal feed supplement.

### 4.1. Fatty Acid Profile of Chlorella vulgaris

*Chlorella vulgaris* has a diverse fatty acid (FA) composition, including saturated fatty acids (SFAs), monounsaturated fatty acids (MUFAs), and polyunsaturated fatty acids (PUFAs) (Table 3). The specific FA profile can vary significantly depending on growth conditions and extraction methods. Palmitic acid (C16:0) is often the most abundant SFA in CLV, with one study reporting it at 59.85% of total FAs [58]. Other significant SFAs include myristic acid (C14:0) and stearic acid (C18:0) [44,56,58]. Lauric acid (C12:0) is present at 0.87% and 6.78% in different studies, and stearic acid (C18:0) ranges from 1.35% to 15.27% (Table 3). Oleic acid (C18:1) is the major MUFA, with reported percentages ranging from 2–51% across different studies [44]. Other MUFAs like palmitoleic acid (C16:1) and hexadecadienoic acid (C16:2) are also present. MUFAs contribute to cell membrane fluidity, which is essential for overall health and metabolic functions [56].

Linoleic acid (C18:2n-6), an omega-6 fatty acid, and alpha-linolenic acid (C18:3n-3), an omega-3 fatty acid, are the predominant PUFAs in CLV (Table 3). CLV is a source of omega-3 fatty acids, including alpha-linolenic acid (ALA), eicosapentaenoic acid (EPA), and docosahexaenoic acid (DHA) [44,55]. However, the levels of these fatty acids vary greatly; for example, DHA content ranges from 0.30% to 20.94% across different studies. Linoleic acid ranges from 9.73% to 26.37%, and elaidic acid (C18:1n-9) at 33.14%. The ratio of omega-3 to omega-6 fatty acids in CLV was found to be 3.00, which is considered beneficial for cardiovascular health and reducing inflammation [55,56]. The fatty acid profile of CLV is highly variable due to factors such as cultivation conditions, harvesting time, and analytical methods. Specific growth conditions like light intensity, temperature, and nutrient availability significantly influence the types and amounts of FAs produced. Further research is needed to optimize the cultivation of *Chlorella vulgaris* to maximize the yield of beneficial fatty acids, especially the essential omega-3s.

### 4.2. Mineral Composition of Chlorella vulgaris

*Chlorella vulgaris* exhibits significant potential as a nutritious feed supplement for animals. However, studies reveal considerable variation in its mineral composition (Table 4), highlighting the need for more research and a deeper understanding of the factors influencing its nutritional content [5]. Phosphorus and potassium are among the most abundant minerals found in CLV. Reported values for phosphorus range from 6500 to 27,080 mg/kg. Potassium levels show even greater variability, ranging from 499.2 to 132,950 mg/kg (Table 4). Sodium content also varies considerably, with reported values between 3820 and 16,450 mg/kg. Tokuşoglu and Üunal [55] reported the following mineral concentrations in mg/kg: sodium (Na) 13,464, phosphorus (P) 17,615, calcium (Ca) 5937, and potassium (K) 499.2. Zheng et al. [27] found phosphorus at 6500 mg/kg and calcium at 2000 mg/kg. Sodium, potassium, magnesium, and iron were not reported in this study. Sucu [48] reported the following mineral concentrations in mg/kg: sodium 16,450, phosphorus 27,080, calcium 940, potassium 132,950, magnesium 12,360, and iron 5400. Martins et al. [3] found the following mineral concentrations in mg/kg: sodium 3820, phosphorus 20,400, calcium 7030, and potassium 29,200. Magnesium and iron were not reported in this study. These variations in mineral content can be attributed to different factors such as the cultivation conditions, analytical techniques used, and the specific strain of Chlorella. These factors directly influence the nutritional value of CLV as a feed supplement.

## 5. *In Vitro* Ruminal Fermentation Parameters

*Chlorella vulgaris* demonstrates a dose-dependent influence on *in vitro* ruminal fermentation, with higher inclusion levels generally leading to more pronounced effects. The impact of CLV on ruminal parameters varies across studies (Table 5), highlighting the importance of dosage and other factors such as basal substrate composition. Studies using lower CLV inclusion levels (0.5–10%) typically report no significant effects on key ruminal fermentation parameters (Table 5). These parameters include total gas production, methane production, total volatile fatty acid (VFA) concentration, and molar proportions of individual VFAs such as acetate, propionate, and butyrate. For example, Gadzama et al. [6] and Vargas et al. [43] observed no effect of CLV on total gas production at these low inclusion rates. However, Kholif et al. [60] reported a significant increase in gas production with 1% CLV inclusion, suggesting variability in responses even at low dosages. Conversely, higher CLV inclusion levels, such as 25%, can lead to decreases in gas production, total VFA concentration, and acetate proportions (Table 5). Sucu [48] observed a decrease in gas production with 25% CLV inclusion. While some studies using low inclusion levels show no effect, both low (1%) and high (25%) CLV inclusion levels have been shown to reduce methane production. Gadzama et al. [6] observed no significant effect on methane production with CLV inclusion at 0.5 and 1%. Vargas et al. [43] also reported no significant effect on methane production, or VFA proportions with 1, 5, and 10% CLV. Kholif et al. [60] found that 1% CLV reduced methane production, and increased ammonia-N and NDF digestibility. Sucu [48] found that 25% CLV decreased methane production, total VFA concentration, and acetate proportions while increasing ammonia-N concentrations.

Differences in basal substrate composition, particularly crude protein (CP) and neutral detergent fiber (NDF) content, may contribute to variations in CLV effects across studies. For example, Kholif et al. [60] used a substrate with 16% CP and 33% NDF, while Vargas et al. [43] used a substrate containing 10% CP and 38% NDF. The dose-dependent nature of CLV’s impact is a critical factor, with low and high inclusion levels producing contrasting outcomes. Recent studies have demonstrated that the inclusion of *Moringa oleifera* can enhance the positive effects of CLV, such as methane reduction. Kholif et al. [60] showed that a combination of CLV (up to 3%) and *Moringa oleifera* reduced methane emissions and protozoal populations, although a minor decrease in overall gas production was also observed. More research is needed to fully understand the effects of CLV on ruminal fermentation, the mechanisms of action, optimal dosages, and potential interactions with different dietary substrates. Future studies should focus on *in vivo* trials to validate these *in vitro* findings and assess the practical applicability of CLV as a feed supplement in ruminant nutrition.

## 6. *Chlorella vulgaris* as Feed Supplement for Cattle

Supplementing cattle diets with CLV has demonstrated inconsistent effects on growth and health across different life stages and breeds (Table 6), with some studies showing decreased daily feed intake in Holstein calves [61], while others report increased feed intake in multiparous Friesian cows [62]. CLV supplementation has been observed to impact milk composition, with some studies reporting increases in milk protein and non-fat solids [54], and in other cases, increases in protein, fat, and iodine concentrations [63]. However, it is important to note that there have been no significant changes in milk production or fat content in some studies [54,63]. The inclusion of CLV in cattle diets may lead to a higher ciliate protozoa population in the rumen [64], with increases in specific genera such as *Isotricha*, *Dasytricha*, *Charonina*, *Buetschlia*, *Ostracodinium*, and *Ophryoscolex* [64]. However, a study by Kuzmaitė et al. [65] found no significant difference in the microflora of neonatal calves. It is crucial to recognize that the effects of CLV supplementation on cattle performance may vary depending on factors such as dosage, form of CLV, and breed of cattle. More so, CLV’s impact on feed intake and efficiency requires further investigation, as studies have shown varying results (Table 6). For example, Shams et al. [62] noticed a decrease in feed efficiency per kg of 4% fat-corrected milk in cows, along with a significant decrease in DMI, total digestible nutrients, crude protein, and digestible crude protein. The overall effects of CLV on cattle performance appear to be complex, thus requiring more research to fully understand its benefits and limitations.

## 7. Impact of *Chlorella vulgaris* on Sheep

**Figure 2:**
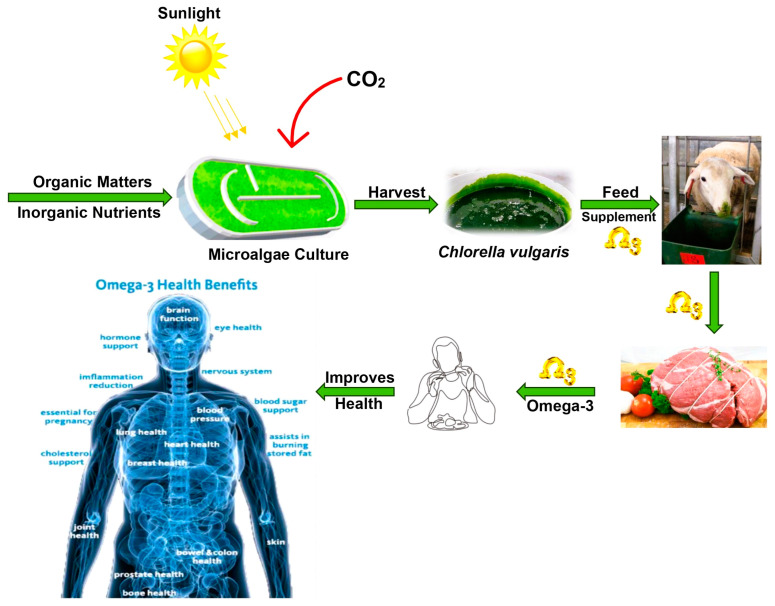
*Chlorella vulgaris* supplementation is a promising strategy to produce value-added meat products for consumers looking to increase their omega-3 intake. Adapted from Gadzama [56].

There is a limited number of *in vivo* studies focused on CLV supplementation in sheep diets, making it challenging to draw firm conclusions about its overall efficacy in this species. However, the few studies reported on the *in vivo* effects of CLV supplementation in sheep, as summarized in Table 7, reveal some potential benefits of CLV supplementation in sheep, though the specific outcomes vary. Gadzama et al. [5] investigated the effects of fresh CLV at 0, 0.5, and 1% of dry matter in 4-month-old lambs, finding a 15.91% decrease in butyrate levels but no significant difference in dry matter intake, feed conversion efficiency, average daily gain, and total weight gain. In contrast, Rabee et al. [66] examined a combination of CLV with yeast (25% *S. cerevisiae*, 50% *S. platensis*, and 25% CLV) at 0 and 1% of dry matter in 5-year-old rams, reporting a 31% increase in dry matter intake (DMI), a 24% decrease in acetate, an 88% increase in propionate, a 16% decrease in butyrate, a 109% increase in isobutyrate, and a 14% increase in neutral detergent fiber (NDF) digestibility. Meanwhile, Slyusarenko et al. [67] studied the effects of CLV suspension at 0, 3, 5, 7, and 9 mL/kg body weight in lactating ewes, with performance measured in lambs, and observed a 66% increase in DMI and a two-fold increase in average daily gain (ADG). Supplementation with fresh CLV microalgae has been shown to enhance the nutritional profile of lamb meat, primarily by increasing beneficial omega-3 fatty acids, with potentially positive implications for human health (Figure 2) [5]. Specifically, the study revealed that incorporating 0.5% dry matter of CLV into the feedlot diet of lambs significantly elevated the concentration of alpha-linolenic acid (ALA) and total omega-3 long-chain fatty acids in the *longissimus lumborum et thoracis* (LTL) muscle. This finding is particularly significant because humans cannot synthesize omega-3 polyunsaturated fatty acids de novo and must obtain them from dietary sources such as eicosapentaenoic acid (EPA) and docosahexaenoic acid (DHA), which are known to reduce the risk of coronary heart disease, neurodegenerative disorders, and metabolic syndromes [5,13,56]. Although the study did not observe a significant increase in EPA and DHA levels in the lamb meat, the elevated ALA content—a precursor to EPA and DHA—still contributes to a more favorable fatty acid profile, which is beneficial for human health (Figure 2) [5,56]. These findings indicate that while CLV supplementation can enhance various performance parameters, the specific effects are influenced by the form and dosage of CLV, as well as the animal’s stage, necessitating further research to optimize its use across different sheep demographics [5,66,67]. Future research should prioritize bridging the gaps between *in vitro* and *in vivo* findings through standardized methodologies, longer-term trials, and exploration of synergistic effects. Additionally, it should focus on the potential to further elevate EPA and DHA levels in meat, thereby maximizing its health benefits for consumers.

## 8. *Chlorella vulgaris* in Goat Diets

Supplementation with CLV in goat diets has revealed several beneficial effects on milk production, nutrient digestibility, and reproductive performance (Table 8). A 10 g/day dose of CLV, in fresh, dried, or lyophilized form, has been shown to increase milk production by 10–12% [22,68]. In addition to milk yield, this dose can enhance DMI by 13% and neutral detergent fiber digestibility by 5% [22]. Furthermore, CLV interacts synergistically with copper sulfate, leading to a 9% increase in NDF digestibility when combined [22,68]. When combined with *Nigella sativa* in pregnant does, CLV has been shown to increase milk yield by 4% and serum antioxidant levels by 22% [69]. Additionally, 5 g/day of CLV with vitamin C improved serum antioxidant levels by 10% in breeding bucks [70].

CLV supplementation has also demonstrated a positive impact on reproductive parameters by improving sperm quality and reducing sperm abnormalities in bucks [70]. A higher dose of 20 g/day of CLV can improve embryo quality and mitochondrial functionality, while 10 g/day supports ovarian follicular growth [71,72]. Moreover, CLV supplementation can improve thermoregulation in lactating goats, reducing rectal temperature and pulse rate under heat stress conditions [73]. In terms of milk composition, CLV supplementation, especially at 10 g/day, has been found to increase monounsaturated fatty acids and conjugated linoleic acid, while reducing saturated fatty acids [29,68,74]. CLV can also increase the milk’s antioxidant levels; for example, an increase in superoxide dismutase by 68% [50]. Table 8 summarizes various studies which confirm the benefits of CLV supplementation in goats, such as increased milk yield and serum antioxidant levels, improved dry matter intake, and enhanced digestibility. However, some studies have shown a reduction in milk yield and an increase in omega-6 [74]. Generally, CLV supplementation at various doses has been shown to increase polyunsaturated fatty acids and omega-3 in milk [29].

**Table 8:** Summary of studies on *Chlorella vulgaris* supplementation in goats.

Studies	CLV, g DM/d	Stage	Dose	Production			Ruminal Fermentation			Digestibility		Anti- Oxidant ^a^	Milk FA
				DMI	ADG	MY	VFA	A:P	NH_3_-N	DM	NDF		
[69]	10 g (and/or 5 g *N. sativa*)	Pregnant	10 g with 5 g *N. sativa*	-	-	+4% ↑			-	+7% ↑	-	+22% ↑	
[70]	5 g (and/or 2 g Vit C)	Breeding buck	5 g with 2 g Vit C		+27% ↑							+10% ↑	
[22]	5 g and 10 g	Lactating	10 g	+13% ↑		+12% ↑	↑	↓	↓	+3% ↑	+5% ↑		PUFA ↑
[68]	5 g, 10 g and 15 g (with CuSO_4_)	Lactating	10 g with CuSO4	-		+10% ↑	↑	-	-	+10% ↑	+9% ↑		MUFA ↑
[74]	10 g	Lactating	-			↓							ω-6 ↑
[29]	5 g and 10 g (dried)	Lactating	10 g (Linear response)										PUFA ↑ ω-3 ↑

^a^ serum antioxidant level; “-”, “↑”, “↓” indicates no difference, increase, and decrease, respectively (*p* < 0.05). DMI = dry matter intake; ADG = average dairy gain; MY = milk yield; VFA = volatile fatty acid; A:P = acetate: propionate; NH_3_-N = ammonia nitrogen; DM = dry matter; NDF = neutral detergent fiber; FA = fatty acid; PUFA = polyunsaturated fatty acid; MUFA = mono-unsaturated fatty acid; ω = omega.

## 9. The Effects of *Chlorella vulgaris* in Broiler Diets

The dietary supplementation of broiler feed with CLV has been extensively researched for its potential to enhance growth performance, health biomarkers, and meat quality (Table 9). Studies have consistently shown that broilers fed CLV at various concentrations and durations exhibit higher growth rates and body weights compared to the control groups. For instance, broilers receiving 1 g of CLV/kg of diet for 32 days showed increased final body weight and weight gain [58], and similar results were observed with 2.5% dried CLV for 8 weeks [75], and with 0.8% CLV for 35 days [21]. Despite the variable incorporation levels, CLV improved broiler growth rate, even at a lower rate of 0.8%. Moreover, improvements in weight gain have been observed with different forms of CLV, including powder, liquid, and *Chlorella* growth factor [34,76,77]. Interestingly, the inclusion of 1.0% *E. coli* fermented liquor with *Chlorella* not only increased weight gain but also improved the feed conversion ratio [78]. However, research indicates that higher inclusion rates of CLV (10–20%) can lead to reduced feed intake and, in some cases, lower body weight and weight gain, suggesting that there may be an optimal level for CLV inclusion to maximize benefits [26,51]. While some studies have shown similar feed intake and feed conversion ratios regardless of CLV inclusion [58,77,79], others have shown improved FCR [21,78], indicating that the effect of CLV on feed efficiency may depend on the concentration and context of its inclusion.

Beyond growth performance, CLV has shown the ability to enhance metabolic health indicators and reduce inflammatory responses in broiler chickens (Table 9). Studies have shown that broilers fed CLV had decreased levels of total lipids in their serum, and lower levels of haptoglobin and interleukin-13, indicating its anti-inflammatory effects [21,34]. Further, CLV supplementation has been associated with improved immunity, as evidenced by increased serum IgG and IgM levels, as well as enhanced gut barrier function through the improved distribution of immune cells and integrity of the intestinal barrier [34,36]. Moreover, CLV is recognized for its ability to regulate the gut microbiota, increasing beneficial bacteria while inhibiting harmful ones, which is believed to contribute to improvements in growth performance and feed efficiency [37,80,81]. Specifically, CLV has been shown to increase *Lactobacillus* populations, and other beneficial bacterial taxa, such as *Clostridium* ASF356 and *Coriobacteriaceae* CHKCI002, enhancing the microbial diversity of the gut [76,82]. The use of CLV as a natural feed additive in broiler diets could contribute to sustainable poultry production, as it promotes healthier birds and safer products for consumers (Table 9).

**Table 9:** Key Findings of *Chlorella vulgaris* supplementation on broiler chicken performance and health.

Summary of Main Findings	References
Broilers fed 1 g of CLV/kg diet for 32 d showed an increase in final body weight and weight gain as compared to control groups	[58]
Broilers fed 2.5% dried *Chlorella vulgaris* for 8 weeks had higher body weight in comparison to the control	[75]
Broilers fed with 0.8% CLV showed better final weight and feed conversion after 35 d than the control group	[21]
Birds fed *Chlorella*, in powder, *Chlorella* growth factor, or liquid form, gained more weight by 5 weeks of age than the control group, without affecting feed intake or efficiency	[76]
Chicks fed 0.05–0.5% *Chlorella* for 35 d had greater weight gains than the control group	[34]
Broilers fed CLV over 5 weeks had significantly higher body weight gain as compared to the control group	[77]
Broilers fed 1.0% *E. coli* fermented liquor with *Chlorella* showed a 2.6% higher weight gain and a 2.8% improved FCR than the control group	[78]
Broilers fed CLV over 5 weeks had similar feed intake and FCR	[77]
Broilers fed 10% CLV for 14 d showed similar growth and feed conversion rates, with or without added CAZymes	[79]
Broilers given 0, 10, or 20 g/kg of CLV had lower feed intake, yet their weight gain and FCR were similar	[26]
Birds on 15% and 20% CLV diets had reduced body weight, weight gain, and feed intake, unlike those on CLV10%, which were comparable to the control group	[51]
Birds fed with 15% and 20% CLV diets showed similar feed conversion ratios to the control group	[51]
Broilers receiving 1 g of CLV/kg of diet had similar feed intake and FCR as compared to the control groups	[58]
Adding 10% CLV and CAZymes to broiler diets did not significantly affect weight gain or feed efficiency	[83]
Chicks fed with 0.15% or 0.5% *Chlorella* or *Chlorella* growth factor showed increased serum IgG and IgM levels compared to the control group	[34]
Chicks fed 0.5% dried *Chlorella* powder had lower serum total lipid concentrations compared to the control	[34]
Broilers fed 0.8% CLV for 35 d showed reduced haptoglobin and interleukin-13 levels compared to the control	[21]
Supplementing broiler diets with 10 g/kg of *Chlorella* by-product enhances health, immunity, antioxidant capacity, and gut morphology	[26]

CLV = *Chlorella vulgaris*; FCR = Feed conversion ratio; d = day/s; CAZymes = carbohydrate-active enzymes.

## 10. Impact of *Chlorella vulgaris* on Meat Quality Parameters of Broiler Chickens

The inclusion of CLV in broiler chicken diets has been shown to enhance meat quality through several mechanisms, including improved nutrient absorption via maintaining the structure of the jejunum [21], and increased breast muscle yield [51], which is associated with higher concentrations of CLV in the diet (Table 10). Furthermore, CLV increases the levels of beneficial fatty acids such as DHA, EPA, and omega-3, leading to a healthier omega-6 to omega-3 ratio, while also boosting levels of carotenoids and antioxidants in the meat, and consequently improving meat color and shelf life [51,79,83]. Additionally, a 20% CLV diet has been found to improve the meat’s water-holding capacity, reduce cooking loss, and make the meat more tender and juicier [34,51,58,83], while also contributing to its oxidative stability through the reduction of harmful compounds, lowered bacterial counts, and increased superoxide dismutase (SOD) activity [58]. Studies have also revealed that meat from chickens fed with 10% CLV had higher consumer acceptance ratings, which suggests that CLV can be included without negatively impacting the taste or overall quality and that it also leads to a reduction in HDL-cholesterol levels in broiler meat [51,79].

## 11. Impact on Laying Hen Performance and Egg Quality

Studies have explored the benefits of supplementing laying hen diets with CLV (Table 11), with research indicating that a 75 g/kg inclusion of CLV can improve both feed intake and egg production [84]. However, other studies have shown that lower levels of CLV, such as 5 g/kg, do not significantly impact the laying performance but can increase egg weight and production [36,85]. The addition of CLV can also improve gut health by increasing the diversity of beneficial bacteria in the gut, which can enhance nutrient absorption and immunity [25]. Further research revealed that CLV can positively affect liver fat content and gut bacteria profiles [27], while fermented CLV is associated with increased egg production, improved yolk color, and enhanced egg freshness [27,36]. Moreover, CLV supplementation has been shown to improve the color parameters of both fresh and boiled eggs, which makes them more appealing to consumers (Table 11). This is because CLV enhances the fatty acid content and ß-carotene concentration in eggs, which can improve the nutritional profile of the eggs [85]. Importantly, studies have found that CLV supplementation does not alter the total cholesterol content in egg yolks [86], which is good news for health-conscious consumers. Furthermore, CLV supplementation has been found to change the fatty acid composition of egg yolks by increasing palmitic and linoleic acids and decreasing docosatetraenoic acid levels [86]. While some studies reported that 5 g of CLV per kg of diet did not affect egg freshness or eggshell quality [36], other studies show that fermented CLV and dietary CLV significantly influenced egg yolk color [85]. Most studies focus on broilers or layers, not breeders. The long-term effects of CLV on reproductive cycles including embryo development, chick vitality, or growth remain understudied.

## 12. Impact of *Chlorella vulgaris* on Pig

Studies on pigs showed that adding CLV to their diet can have varied effects, with most studies indicating that it does not significantly change growth parameters like average daily gain, average daily feed intake, and feed conversion ratio (Table 12). For instance, a 5% CLV diet led to increased feed intake in weaned piglets, but it did not affect final weight or growth rate [3,87], and similarly, other studies using 1% and 5% CLV found no differences in growth performance [45,88,89]. However, fermented CLV at 0.1% has been shown to improve ADG and dry matter digestibility, suggesting potential probiotic benefits [90]. CLV has also demonstrated some immune and metabolic impacts, including an increase in immunoglobulin G levels and a decrease in immunoglobulin M levels, but it can also increase total cholesterol, LDL, and VLDL cholesterol while decreasing HDL cholesterol [3,47]. Although CLV does not significantly impact meat quality [47], it can increase antioxidant pigments and omega-3 fatty acids in pork fat, along with decreasing the ratio of omega-6 to omega-3 fatty acids in the liver and pork [3,47,87,89]. Overall, CLV supplementation can improve fatty acid profiles and increase beneficial pigments; however, it is important to carefully manage its levels in pig diets due to potential negative effects on the immune system (Table 12).

## 13. *Chlorella vulgaris* in Rabbit Diets

Research on supplementing rabbit diets with CLV has shown varied results regarding growth and feed efficiency, with some studies reporting improved growth and feed-to-gain ratios at CLV levels of 200–500 mg/kg of body weight, while others found no significant differences in these parameters (Table 13). Specifically, Sikiru et al. [91] found that rabbits fed 300 and 500 mg/kg of CLV showed higher final weight and weight gain, while another indicated that 500 mg/kg of CLV led to decreased feed consumption [92]. Additionally, CLV supplementation has been associated with reduced oxidative stress, indicated by lower levels of malondialdehyde and protein carbonyl concentrations, and increased antioxidant levels [91,93]. Moreover, rabbits given CLV exhibited improved immune health, with higher levels of immunoglobulins (IgG and IgM) increased glutathione activity, and better lipid profiles, with reduced serum triglycerides and low-density lipoprotein levels, thus showing potential cardiovascular benefits in rabbits [91,93]. However, CLV did not significantly affect nutrient digestibility or dressing percentages [94].

## 14. Impact of *Chlorella vulgaris* on Fish

*Chlorella vulgaris* is a promising functional feed additive for aquaculture due to its rich nutritional profile and bioactive compounds, which have been shown to improve growth, feed utilization, and overall health in various fish species (Table 14). Studies have shown that dietary inclusion of CLV enhances feed palatability, likely due to amino acids, peptides, and nucleotides that act as flavor enhancers, which leads to increased feed intake and improved nutrient digestibility [95,96]. Several studies have explored the effects of different levels of CLV inclusion on fish diets, with optimal levels varying depending on the species [97,98,99]; for instance, 5% CLV in Nile tilapia diets has been shown to improve growth and feed efficiency [100], while levels up to 15 g/kg have also shown positive effects [96]. CLV has been shown to significantly improve growth rates and feed conversion ratio in several species, including tilapia [96,100], seabass [33], and common carp [101], as well as other aquatic species (Table 14), with some studies even observing a 70% increase in final body weight in juvenile seabass [33]. Additionally, CLV has demonstrated positive impacts on immunity and disease resistance, with lipopolysaccharides and carotenoids stimulating immune responses and providing protection against bacterial pathogens in several species [102,103]. Furthermore, CLV’s antioxidant properties and ability to manage stress have been observed, as it modulates key antioxidant enzymes such as Superoxide Dismutase, Catalase, and Glutathione Peroxidase, and mitigates the negative effects of stress [39,104,105]. However, it’s important to note that while CLV generally enhances growth and health, some studies have reported varied results, such as similar growth, feed conversion, and protein efficiency in seabreams when using CLV as a fishmeal substitute [106]. Others reported that a 5% CLV diet resulted in similar SGR, FCR, and PER values as the control group in one case [100], and some studies have shown that high levels (75%) of CLV in the diet can lead to decreased feed consumption [107]. Our findings demonstrate that CLV exhibits dual prebiotic and phytogenic effects, enhancing the growth and viability of probiotic bacteria while showing potential inhibitory activity against intestinal pathogens. These results suggest that CLV could serve as a multifunctional feed supplement, leveraging its prebiotic properties to support beneficial gut microbiota and its phytogenic properties to mitigate pathogenic infections. However, the precise mechanisms underlying these effects remain incompletely understood. Further research is warranted to elucidate the specific pathways through which CLV functions as a prebiotic, probiotic, and/or phytogenic agent across diverse animal species. Such investigations will be critical for optimizing its application in animal nutrition and for validating its potential to improve gut health and overall productivity in livestock.

## 15. Conclusions

*Chlorella vulgaris* shows promise as a beneficial ingredient in animal diets, with studies indicating improvements in growth performance, health outcomes, and product quality when used at appropriate inclusion rates. However, due to variability in responses across species and inclusion levels, species-specific dietary formulations are necessary. Further research is needed to optimize CLV inclusion rates, maximizing benefits while minimizing potential drawbacks. Therefore, CLV represents a valuable alternative to traditional feed ingredients, which could significantly contribute to more environmentally friendly and resilient livestock production systems.

## Figures and Tables

**Table 1:** Some *Chlorella vulgaris* (CLV) production companies as reported in various studies.

Source	Location	References
Cultured CLV biomass produced by Phycom, Utrecht	The Netherlands	[21]
CLV produced by the Algal Biotechnology Unit, National Research Centre, Giza	Egypt	[22,23]
Chlorella (UTEX 2805) from Aqualgae’s vertical photobioreactor in Almerìa	Spain	[24]
CLV grown in a photobioreactor with sunlight at IGV, Nuthetal	Germany	[25]
Dried CLV biomass from SurNature Biological Technology Co., Ltd., Xi’an	China	[26]
Fresh CLV cultivated by Genesis Co. Pty Ltd., Bowen	Australia	[6]
A commercial product of CLV produced by fermentation (CBT^®^), Celltech Co., Ltd., Eumseong-gun	Republic of Korea	[27]
CLV cultivated in a photobioreactor at Athens’ Agricultural University’s Molecular Biology Lab., Athens	Greece	[28]
CLV (Beij., 1996/H 14) produced in the laboratory of the Institute of Botany, Třeboň	Czech Republic	[29]
Dried CLV produced by commercial algae culture in Menoufia governorate	Egypt	[30]
CLV meal produced by Demeter Bio-Tech Co., Ltd., Wuhan	China	[31]
CLV cultured from the algal culture unit of CIFA, Bhubaneswar	India	[32]
Commercial CLV (Algaessence^®^ feed) by ALGAplus, Ílhavo and Allmicroalgae, Pataias	Portugal	[33]
Dried CLV powder and *Chlorella* Growth Factor from Daesang Corp., Icheon	Republic of Korea	[34]
Dry CLV powder produced by Daesang Corporation, Seoul	Republic of Korea	[35]
CLV strain (PKVL7422) from Korean Collection for Type Cultures (13361BP), Daejeon	Republic of Korea	[36]
CLV from FACHB’s Freshwater Algae Collection, Wuhan	China	[37]
CLV produced by NEOALGAE, Gijón, Asturias	Spain	[38]
Dried CLV powder produced by the Institute of National Research Center, Cairo	Egypt	[39]
CLV powder Organic Traditions Company, Advantage Health Matters Inc., Toronto, ON	Canada	[40]
CLV cultivated from Baton Rouge, Louisiana	USA	[41]
CLV strain (SAG 211–12) grown in 500 mL flasks on an orbital shaker (KS 501 digital, Ika-Werke) in Staufen	Germany	[42]
Dried and pelleted CLV produced by Origo, LLC, Venus, FL	USA	[43]
CLV from UTEX Algae Collection, Algoteca, University of Texas, Austin, TX	USA	[44]
CLV powder from Setalg©, Pleubian	France	[45]
CLV processed by Algosource Technologies, Saint-Nazaire	France	[46]
CLV produced by Allmicroalgae (Natural Products, Portugal), Leiria	Portugal	[47]
CLV cultivated in a flat panel photobioreactor under controlled conditions without any contamination	Turkey	[48]
CLV strain (CCAP 211) from the Culture Collection of Algae and Protozoa, Argyll	UK	[49]

**Table 3:** Fatty acid profile of *Chlorella vulgaris*.

Fatty Acids	% of Total Fatty Acids	Reference
Butyric (C4:0)	0.20	[59]
Caproic (C6:0)	2.77	[59]
Caprylic (C8:0)	0.26	[59]
Undecanoic (C11:0)	1.39	[59]
Undecenoic (C11:1)	2.17	[59]
Lauric acid (C12:0)	0.87 6.78	[59] [44]
Lauroleic (C12:1)	0.41	[59]
Tridecanoic (C13:0)	1.03	[59]
Myristic acid (C14:0)	0.38 0.69 1.13 6.91 15.90	[55] [59] [3] [58] [44]
Pentadecanoic (C15:0)	1.70	[59]
Pentadecenoic (C15:1)	3.53	[59]
Palmitic acid (C16:0)	14.42 15.41 17.20 20.90 37.10 59.85	[59] [55] [3] [44] [50] [58]
Palmitoleic (C16:1)	1.17 3.52 3.90 4.04 14.30	[55] [58] [3] [59] [44]
Hexadecadienoic (C16:2)	5.34	[59]
Hexadecatrienoic (C16:3)	4.90	[59]
Margaric acid (C17:0)	0.12 0.23	[59] [3]
Heptadecenoic (C17:1)	0.27 0.61	[59] [3]
Stearic acid (C18:0)	1.35 1.57 3.00 6.24 13.40 15.27	[50] [59] [3] [55] [44] [58]
Oleic acid (C18:1)	2.43 6.36 11.70 17.62 50.90	[50] [58] [3] [59] [44]
Vaccenic acid (C18:1n-7)	1.13	[55]
Elaidic acid (C18:1n-9)	33.14	[55]
Linoleic acid (C18:2n-6)	9.73 11.20 11.97 12.30 22.04 26.37	[55] [3] [59] [44] [50] [58]
Alpha-Linolenic acid (C18:3n-3)	1.93 10.10 11.82 15.79 19.10 22.10	[55] [3] [58] [59] [44] [50]
Arachidic acid Eicosanoic (C20:0)	0.14 0.17 0.19 26.22	[3] [59] [55] [58]
Gondoic acid (C20:1)	0.13	[3]
Eicosapentaenoic acid-EPA (C20:5n-3)	1.26 1.61 3.23 ND ^a^	[58] [44] [55] [59]
Lignoceric acid (C22:0)	0.06	[3]
Docosapentaenoic-DPA (C22:5n-3)	3.11	[55]
Docosahexaenoic acidDHA(C22:6n-3)	0.30 1.99 20.94	[59] [58] [55]
Tetracosanoic (C24:0)	0.22	[59]
∑ SFA	22.22	[55]
∑ MUFA	35.44	[55]
∑ PUFA	38.94	[55]
∑ n-3	29.21	[55]
∑ n-6	9.73	[55]
∑ n-3/n-6	3.00	[55]
DHA/EPA	6.73	[55]

^a^ = not detected; n, omega; ∑, summation of; SFA, saturated fatty acid; MUFA, monounsaturated fatty acid; PUFA, polyunsaturated fatty acid; n-3, omega-3 fatty acid; n-6, omega-6 fatty acid.

**Table 4:** Analysis of *Chlorella vulgaris* mineral content across different studies.

	Tokuşoglu and Üunal [55]	Zheng et al. [27]	Sucu [48]	Martins et al. [3]
Indices	mg/kg	mg/kg	mg/kg	mg/kg
Na	13,464	NR	16,450	3820
P	17,615	6500	27,080	20,400
Ca	5937	2000	940	7030
K	499.2	NR	132,950	29,200
Mg	3443	NR	12,360	NR
Fe	2591	NR	5400	NR
Cr	0.2	NR	NR	NR
Cu	0.6	NR	0	NR
Zn	11.9	NR	530	NR
Mn	20.9	NR	1270	NR
Se	0.7	NR	NR	NR
B	NR	NR	1640	NR

NR, not reported; Na, Sodium; P, Phosphorus; Ca, Calcium; K, Potassium; Mg, Magnesium; Fe, Iron; Cr, Chromium; Cu, Copper; Zn, Zinc; Mn, Manganese; Se, Selenium; B: Boron.

**Table 5:** Studies on the effects of *Chlorella vulgaris* (CLV) on *in vitro* ruminal fermentation.

Parameters	Gadzama et al. [6] ^a^	Vargas et al. [43] ^b^	Kholif et al. [60] ^c^	Sucu [48] ^d^
CLV (% DM)	0, 0.5, and 1%	0, 1, 5, and 10%	0, 1, 2 and 3%	0 and 25%
Incubation time	24 h	24 h	48 h	48 h
Key findings				
Total gas production	No effect	No effect	1% CLV ↑	25% CLV ↓
Methane production	No effect	No effect	1% CLV ↓	25% CLV ↓
Total VFA	No effect	No effect	No effect	25% CLV ↓
% of VFA				
Acetate	No effect	No effect	No effect	25% CLV ↓
Propionate	No effect	No effect	No effect	No effect
Butyrate	No effect	No effect	No effect	No effect
Ammonia-N	N/A	No effect	1% CLV ↑	25% CLV ↑
NDF digestibility	N/A	N/A	1% CLV ↑	N/A

Basal substrate contained ^a^ 15% CP and 35% NDF; ^b^ 10% CP and 38% NDF; ^c^ 16% CP and 33% NDF; ^d^ two basal substrates, wheat silage (11% CP and 41% NDF) and corn silage (6% CP and 4% NDF), were used, the findings presented in this table as the main effect of CLV. “↑”, “↓” indicate increase, and decrease, respectively (*p* < 0.05); N/A = not reported.

**Table 6:** Studies on the impact of *Chlorella vulgaris* (CLV) on Cattle.

Summary of Main Findings	References
Holstein cows in mid-lactation fed 30 g each of either conventional or lutein-fortified *Chlorella* for 3 weeks showed no significant differences in feed consumption, milk production, or milk fat percentage when compared to a control group	[54]
Cows fed *Chlorella* showed increased concentration of milk protein and non-fat solids than those in the control group	[54]
Milk from cows fed lutein-enriched *Chlorella* showed higher lutein levels than milk from cows fed standard *Chlorella* or control	[54]
Feeding Holstein heifer calves *Chlorella* spp. (60 g/d) led to a decrease in their daily feed intake compared to calves that did not receive *Chlorella*	[61]
Holstein cows receiving 1 to 1.5 L of CLV suspension daily showed higher concentrations of protein, fat, and iodine in their milk than the control groups	[63]
Neonate calves fed 400 mL daily of CLV IFR-111 for 30 d showed no significant difference in microflora compared to the control group	[65]
Cows receiving 90 and 170 g of lyophilized CLV in their TMR had a higher ciliate protozoa population after 21 d, surpassing that of the control diet	[64]
Dietary inclusion of CLV at 30, 90, and 170 g per diet enhanced the ruminal protozoa population, boosting genera like *Isotricha*, *Dasytricha*, *Charonina*, *Buetschlia*, *Ostracodinium*, and *Ophryoscolex*	[64]
Multiparous Friesian cows that received 2 mL or 4 mL of CLV per kg BW showed increased feed intake up to day 120 of lactation, compared to cows that did not receive CLV	[62]
For cows fed 2 mL and 4 mL of CLV, feed efficiency per kg for 4% FCM decreased	[62]
Cows fed 2 mL and 4 mL of CLV showed a decrease in DMI by 10.66% and 18.85%, TDN by 8% and 13.33%, CP by 10.17% and 18.55%, and DCP by 7.58% and 13.32%, respectively, compared to the control group	[62]

TMR = total mixed ration; BW = body weight; DMI = dry matter intake; CP = crude protein; TDN = total digestible nutrients; DCP = digestible crude protein; FCM = fat-corrected milk; g = grams; spp. = species; mL = milliliter; d = day/s.

**Table 7:** Studies on *in vivo* effects of *Chlorella vulgaris* (CLV) supplementation in sheep.

Parameters	Gadzama et al. [5]	Rabee et al. [66]	Slyusarenko et al. [67]
CLV form	Fresh (g/100 g DM)	Combination with yeast ^a^ (g/100 g DM)	Suspension (mL/kg of ewe BW)
CLV dose	0, 0.5, and 1%	0 and 1%	0, 3, 5, 7, and 9 mL/kg BW
Stage	Lamb (4 months)	Ram (5 years)	Lactating ewe ^b^
Key findings ^c^			Response in lamb
DMI	-	+31% ↑	+66% ↑
ADG	-		+2X ↑
Total VFA	-	-	
% of VFA			
Acetate	-	−24% ↓	
Propionate	-	+88% ↑	
Butyrate	15.91% ↓	−16% ↓	
Isobutyrate	-	+109% ↑	
Ammonia-N		-	
NDF digestibility		+14% ↑	

DMI = dry matter intake; ADG = average daily gain; VFA = volatile fatty acid; Ammonia-N = ammonia nitrogen; NDF = neutral detergent fiber; DM = dry matter; BW = body weight. ^a^ 25% *S. cerevisiae*, 50% *S. platensis*, and 25% CLV. ^b^ suspension was fed to lactating ewe for first 20 days and performance was measured in lamb. ^c^ key findings were summarized by polled effect of CLV supplementation over control. “-”, “↑”, “↓” indicate no difference, increase, and decrease, respectively (*p* < 0.05).

**Table 10:** Summary of studies on the Effects of *Chlorella vulgaris* on broiler chicken meat quality.

Summary of Main Findings	References
Broilers fed diets containing 10, 15, or 20% CLV showed higher ileal digesta viscosity and greater gastrointestinal size, which also led to increased breast muscle yield	[51]
Feeding CLV to broilers raised levels of DHA, EPA, and n-3 PUFA in their breast meat and lowered the n-6/n-3 PUFA ratio	[51]
Adding 10% CLV and CAZymes to broiler diets increased plasma total lipids and improved the n-6/n-3 ratio and total carotenoids	[79]
Adding CLV led to a yellower breast muscle, significantly boosting chlorophyll a, b, and total carotenoids	[51]
Dietary CLV enhanced the color and carotenoid content in poultry meat	[83]
Broilers given a 20% CLV diet showed improved meat water-holding capacity and reduced cooking loss	[51]
Broilers fed dried *Chlorella* powder and *Chlorella* growth factor had similar meat qualities such as pH, color, and cooking loss	[34]
Broilers fed CLV at 1 g/kg diet showed lower levels of malondialdehyde and protein carbonyl, reduced cooking loss and bacterial counts, and higher SOD activity than the control group	[58]
Dietary CLV reduced bacteria levels in meat in comparison to control groups	[51]
Broilers given 10% CLV in their feed for 2 weeks showed comparable breast muscle quality, tenderness, juiciness, and taste to those on a standard diet	[83]
Broiler breast meat, enhanced with 10% CLV in their feed for 40 d, showed higher acceptance ratings	[51]
CLV supplementation led to lower HDL-cholesterol in broilers’ meat	[79]

CLV = *Chlorella vulgaris*; DHA = docosahexaenoic acid; EPA = eicosapentaenoic acid; HDL = high-density lipoprotein; n-3 PUFA = omega-3 polyunsaturated fatty; n-6 = omega-6 fatty acids; n-3 = omega-3 fatty acids; CAZymes = carbohydrate-active enzymes, d = day/s; SOD = superoxide dismutase.

**Table 11:** Summary of studies on the effects of *Chlorella vulgaris* on laying hen performance and egg quality.

Summary of Main Findings	References
Hen-day egg production and feed intake improved with higher levels of *Chlorella* by-product at 75 g/kg of basal diet	[84]
Lohmann Brown hens fed 2.0% CLV for 8 weeks had similar feed intake, final body weight and cholesterol levels as compared to control	[85]
Hy-Line Brown laying hens fed a 5 g CLV/kg diet had a similar laying performance as the control group	[36]
CLV-supplemented hens had greater egg weight (62.4 g) as compared to (59.8 g) in the control	[85]
Feeding spray-dried or bullet-milled spray-dried CLV at 5.0 g/kg increased the diversity of lactobacilli in the crop of Lohmann Brown laying hens as compared to the control group	[25]
Dietary CLV in the diet of hens resulted in a more diverse bacterial community in the ceca of Lohmann Brown hens	[25]
CLV levels of 0, 1000, or 2000 mg/kg positively affected the contents of hepatic triacylglycerol and the profiles of cecal microflora in Hy-line Brown hens	[27]
Hens fed CLV at 5 g of diet did not differ in laying performance, jejunal histology, cecal short-chain fatty acids, and antioxidant/immune markers in ileal mucosa	[36]
Indicators of egg freshness (Haugh unit) and eggshell quality (i.e., strength and thickness) were not altered by 5 g dietary CLV in the diets of Hy-Line Brown laying hens	[36]
A total of 80 weeks Hy-line Brown layers fed fermented CLV levels of 0, 1000, or 2000 mg/kg diet for 42 d showed a linear increase in egg production, egg yolk color, and Haugh unit	[27]
CLV supplementation improved the color parameters (L*, a*, and b*) of fresh and 10 min boiled eggs compared to the control group	[85]
Dietary CLV significantly influenced egg yolk color in laying hens compared to the control group	[36]
Dietary CLV increased fatty acid content, ß-carotene concentration, antioxidant capacity, yolk color intensity, and boiling eggs enhanced the b* colour	[85]
A total of 74 weeks Bovans Braun laying hens fed *Chlorella* at 2% and 10% of the diet for 37 days had similar total cholesterol content in 100 g of yolk as compared to the control	[86]
*Chlorella* supplementation increased palmitic and linoleic acid concentration but decreased docosatetraenic acid in egg yolk	[86]

CLV = *Chlorella vulgaris*; L* = lightness; a* = red/green; b* = yellowness/blue.

**Table 12:** Summary of studies on the effects of *Chlorella vulgaris* on pig growth performance and health.

Summary of Main Findings	References
Weaned male piglets fed 5% CLV in their diet for 15 d had higher ADFI compared to the control but no changes in final weight, ADG, and FCR	[3]
Pigs fed 0.1% levels of fermented *Chlorella* for 6 weeks had higher ADG and DM digestibility but similar ADFI and G:F ratio as compared to the control group, or the 0.2% CLV-supplemented group	[90]
Growth performance of finishing pigs fed 5% CLV was not different from the control group, receiving a soybean meal-based diet	[89]
Weaned piglets fed 1.0% of CLV for 11 days had similar body weight as compared to the control	[88]
Pigs supplemented with 1% CLV from 28 to 42 d had similar ADFI, ADG, and gain: feed (G:F) ratio compared to control	[45]
Oral administration of CLV to suckling piglets at 385 mg/kg BW per day resulted in a similar post-weaning ADG, feed intake, and G:F ratio	[46]
Post-weaned male piglets fed CLV at 5% levels of diet for 2 weeks had similar growth as the control	[87]
CLV had no significant effect on pigs’ carcass characteristics	[89]
Pigs fed CLV-based diets had increased IgG and decreased IgM levels	[3]
A high dietary level of CLV at 5% of the diet impacts the blood parameters of finishing pigs, with a notable immunosuppressive effect increasing susceptibility to infectious diseases	[89]
Piglets fed CLV levels of 0%, 5%, 5% + Rovabio, and 5% + CAZyme mixture had increased total LDL-, and VLDL-cholesterol while HDL-cholesterol decreased after 15 days	[3]
CLV had no significant effect on pigs’ meat quality traits	[89]
Finishing pigs fed CLV showed increased lipid-soluble antioxidant pigments and n-3 PUFA in pork fat	[89]
Dietary CLV decreased n-6 PUFA, and increased n-3 PUFA, improving the n-6/n-3 ratio in the liver of pigs	[3]
CLV decreased n-6/n-3 fatty acid ratio, improving pork fat nutritional value	[89]
Pigs fed 5% CLV for 2 weeks had greater total carotenoids and n-3 PUFA, and better n-6/n-3 fatty acid ratio	[87]
CLV reduced systemic antioxidant capacity in pigs, and increased hepatic n-3 PUFA content, reducing the n-6/n-3 ratio	[47]

CLV = *Chlorella vulgaris*; ADFI = average daily feed intake; ADG = average daily gain; FCR = feed conversion ratio; BW = body weight; DM = dry matter; G:F = gain to feed ratio; n-3 = omega-3; n-6 = omega-6; PUFA = polyunsaturated fatty acids; LDL = low density lipoprotein; HDL = high-density lipoprotein; VLDL = very low-density lipoprotein.

**Table 13:** Effects of dietary *Chlorella vulgaris* on growth performance and health parameters in rabbits.

Summary of Main Findings	References
Rabbits fed 200–500 mg CLV/kg BW had a higher final body weight and feed-to-gain ratio than the control	[91]
Rabbits showed similar live weight, weight gain, feed consumption, and FCR when given 0.5 g, 1.0 g, or 1.5 g of CLV powder/kg of diet over 8 weeks	[93]
Rabbits fed 300 and 500 mg/kg of CLV for 8 weeks had higher final weight and weight gain than the control	[92]
Rabbits on a diet with 0.75 g/kg of CLV had a better FCR than the control group	[94]
Dietary CLV levels of 300 and 500 mg/kg enhanced FCR as compared to the control group	[92]
New Zealand White rabbits showed no significant differences in feed intake, final body weight, or weight gain when fed diets with 0.75 or 1.5 g/kg of CLV compared to the control group over 12 weeks	[94]
New Zealand White rabbits fed 500 mg/kg BW of CLV biomass showed a decrease in feed consumption relative to the control group	[91]
New Zealand male rabbits fed a high dose of CLV 500 mg/kg diet for 8 weeks had reduced feed intake	[92]
Rabbits fed CLV showed no significant differences in nutrient digestibility (DM, OM, CP, CF, EE, and NFE)	[94]
Rabbits fed CLV levels of 0.75 or 1.5 g/kg diet had similar dressing percentages, liver, kidney, heart, and total giblet weights and percentages compared to the control	[94]
Malondialdehyde levels, an indicator of oxidative stress, were lower in rabbits fed CLV	[93]
CLV levels of 500 mg/kg BW reduced malondialdehyde, and protein carbonyl concentrations compared to the control	[91]
Rabbits fed CLV had improved immunoglobulins (IgG and IgM), and glutathione activities compared to the control	[93]
Rabbits fed a 1.0 g CLV/kg diet had enhanced immune responses and antioxidant status compared to the control	[93]
Rabbits fed CLV levels of 0, 0.5, 1.0, or 1.5 g/kg diet showed reduced serum triglycerides and low-density lipoprotein compared to control after 8 weeks	[93]

CLV = *Chlorella vulgaris*; FCR = feed conversion ratio; BW = body weight; DM = dry matter; OM = organic matter; CP = crude protein; CF = crude fiber; EE = ether extract; NFE = nitrogen-free extract.

**Table 14:** Studies on the impact of *Chlorella vulgaris* on fish performance and health.

Summary of Main Findings	References
Increasing levels of CLV 0, 2.5, 5, 10, 15, and 20 g/kg diet enhanced feed consumption, growth rate, and SGR in Tilapia for 70 d	[96]
Fish fed diets with 5%, 10%, and particularly 15% CLV showed a greater final body weight after 8 weeks, compared to the control group	[30]
Juvenile seabass fed from 0 to 6% CLV blend showed a 70% increase in final body weight compared to the control group over 12 weeks	[33]
Juvenile fish fed 75% CLV at 2% body weight had improved FCR and PER compared to the control group	[107]
Olive flounder had higher growth rates over 8 weeks when fed with increasing levels of CLV 0, 5, 10, and 15%	[108]
Catfish fed 75% CLV diet at 2% BW had higher body weight and SGR compared to the control group	[107]
Largemouth bass showed a quadratic increase in body weight, growth rate, and feed consumption over 8 weeks when fed a diet with 0, 25, 50, 75 and 100% CLV meal as a replacement for fishmeal	[31]
Fingerlings fed increasing levels (0, 0.1, 0.5, and 1%) of CLV per kg diet for 90 d had improved growth rates and FCR as compared to the control	[32]
Nile Tilapia on a 5% CLV diet for 60 days showed improved survival and growth over the control group	[100]
Fish survival rates remained consistent across varying CLV concentrations (0, 5, 10, and 15%)	[108]
Tilapia fingerlings fed diets with CLV levels ranging from 0 to 20 g/kg had comparable FCR and survival rates	[96]
Seabreams given 10–30% CLV in their diet as a fishmeal substitute showed similar growth, feed conversion, and protein efficiency after 12 weeks	[106]
As dietary CL levels rose from 0% to 100%, fish FCR increased linearly	[31]
Common carp given a diet with 5% CLV for 56 d, showed increased final weight and SGR and a reduced FCR, in contrast to the control group	[101]
Tilapia fingerlings receiving a diet with 4.76% CLV powder for 90 d had higher SGR over the control group	[35]
Fish fed with a 5% CLV diet for 60 d, showed SGR, FCR, and PER values like those of the control group	[100]
Rainbow trout fed 0.2% CLV or synthetic carotenoids for 9 weeks had similar feed intake and weight gain	[109]
African catfish fed 50% and 75% CLV for 12 weeks, as a fishmeal substitute at 2% BW showed a decrease in feed consumption	[107]
A diet with 2% and 4% CLV blend decreased fish FCR after 12 weeks	[33]
Common carp fed dietary CLV levels of 5% and 10% showed blood parameters Hb, RBC, and WBC comparable to the control group	[110]
A 5% concentration of CLV enhanced growth, blood health, antioxidant levels, immunity, survival, and gene activity in Nile tilapia, countering the effects of 15 μg/L deltamethrin over 8 weeks	[111]
Dietary CLV resulted in a minor reduction of muscle carotenoid levels in rainbow trout, recording 11.9 mg/kg compared to 13.3 mg/kg observed with synthetic carotenoids	[109]

CLV = *Chlorella vulgaris*; SGR = specific growth rate; FCR = feed conversion ratio; PER = protein efficiency ratio; BW = body weight; d = days; Hb = hemoglobin; RBC = red blood cells; WBC = white blood cells.

## Data Availability

No new data were created or analyzed in this study. Data sharing does not apply to this article.
